# Application of PD-1 Blockade in Cancer Immunotherapy

**DOI:** 10.1016/j.csbj.2019.03.006

**Published:** 2019-05-23

**Authors:** Xiaomo Wu, Zhongkai Gu, Yang Chen, Borui Chen, Wei Chen, Liqiang Weng, Xiaolong Liu

**Affiliations:** a Dermatology Institute of Fuzhou, Dermatology Hospital of Fuzhou, Xihong Road 243, Fuzhou 350025, PR China; b The Institute of Biomedical Sciences, Fudan University, Mingdao Building, Dongan Road 131, Shanghai 200032, PR China; c Department of Biomedicine, University of Basel, Klingelbergstr. 70, CH-4056 Basel, Switzerland; d The United Innovation of Mengchao Hepatobiliary Technology Key Laboratory of Fujian Province, Mengchao Hepatobiliary Hospital of Fujian Medical University, Xihong Road 312, Fuzhou 350025, PR China; e Liver Disease Center, The First Affiliated Hospital of Fujian Medical University, Chazhong Road 20, Fuzhou 350005, PR China

**Keywords:** Immunotherapy, PD-1, PD-L1, Immune surveillance, Checkpoint blockade, NSCLC, Non-small cell lung cancer, RCC, Renal cell carcinoma, cHL, Classical Hodgkin lymphoma, HNSCC, Head and neck squamous cell carcinoma, UC, Urothelial carcinoma, CRC, Colorectal cancer, HCC, Hepatocellular carcinoma, SCLC, Small cell lung cancer, MSI-H, Microsatellite instability-high, dMMR, DNA mismatch repair deficiency, GC, Gastric cancer, CC, Cervical cancer, PMBCL, Primary mediastinal large B-cell lymphoma, MCC, Merkel cell carcinoma, AEs, Adverse events, PD-1, Programmed cell death 1, PD-L1, Programmed death-ligand 1, CTLA-4, Cytotoxic T-lymphocyte–associated antigen 4, ICOS, Inducible T-cell co-stimulator, DCM, Dilated cardiomyopathy, cTnI, Cardiac troponin I, ITIM, Immune-receptortyrosine-based inhibitory motif, ITSM, Immune-receptortyrosine-based switch motif, PTPs, Protein tyrosine phosphatases, SHP2, Src homology 2 domain-containing phosphatase 2, BATF, Basic leucine zipper transcriptional factor ATF-like, ZAP70, Zeta-chain-associated protein kinase 70, PI3K, Phosphoinositide 3-kinase, AP1, Activator protein 1, NFAT, Nuclear factor of activated T cells, LCK, Tyrosine-protein kinase Lck, ERK, Extracellular signal–regulated kinase, PKC, Protein kinase C, TCR, T-cell receptor, NF-κB, Nuclear factor-κB, TIICs, Tumor infiltrating immune cells, TILs, Tumor-infiltrating lymphocytes, TME, Tumor microenvironment, IHC, Immunohistochemistry, IFN, Interferon, ORR, Overall response rate, OS, Overall survival, PFS, Progression-free survival, HR, Hazard ratio, ICC, Investigator-choice chemotherapy, BICR, Blinded Independent Central Review, DOR, Duration overall response, BV, Brentuximab vedotin, ASCT, Autologous stem cell transplantation, GEJ, GASTRIC or gastroesophageal junction, ITT, Intention-to-treat, IrAEs, Immune related adverse events, APCs, Antigen presenting cells, DCs, Dendritic cells, TMB, Tumor mutation burden, MHC, Major histocompatibility, MAP, Mitogen-activated protein, CXCL9, C-X-C motif chemokine ligand 9, TGF, Transforming growth factor, B2M, β2 microglobulin, PTEN, Phosphatase and tensin homolog, VEGF, Vascular endothelial growth factor, PRC2, Polycomb repressive complex 2, JMJD3, Jumonji Domain-Containing Protein 3, EZH2, Enhancer of zeste homolog 2, DZNep, 3-Deazaneplanocin A, DNMT, DNA methyltransferase, 5-AZA-dC, 5-aza-2′-deoxycytidine, LAG3, Lymphocyte-activation gene 3, TIM3, T-cell immunoglobulin and mucin-domain containing-3, SIRPα, Signal-regulatory protein alpha, ADCC, Antibody-dependent cellular cytotoxicity

## Abstract

The programmed cell death protein 1 (PD-1) pathway has received considerable attention due to its role in eliciting the immune checkpoint response of T cells, resulting in tumor cells capable of evading immune surveillance and being highly refractory to conventional chemotherapy. Application of anti-PD-1/PD-L1 antibodies as checkpoint inhibitors is rapidly becoming a promising therapeutic approach in treating tumors, and some of them have successfully been commercialized in the past few years. However, not all patients show complete responses and adverse events have been noted, suggesting a better understanding of PD-1 pathway mediated immunosuppression is needed to predict patient response and improve treatment efficacy. Here, we review the progresses on the studies of the mechanistic role of PD-1 pathway in the tumor immune evasion, recent clinical development and commercialization of PD-1 pathway inhibitors, the toxicities associated with PD-1 blockade observed in clinical trials as well as how to improve therapeutic efficacy and safety of cancer immunotherapy.

## Graphical Abstract

**Figure:**
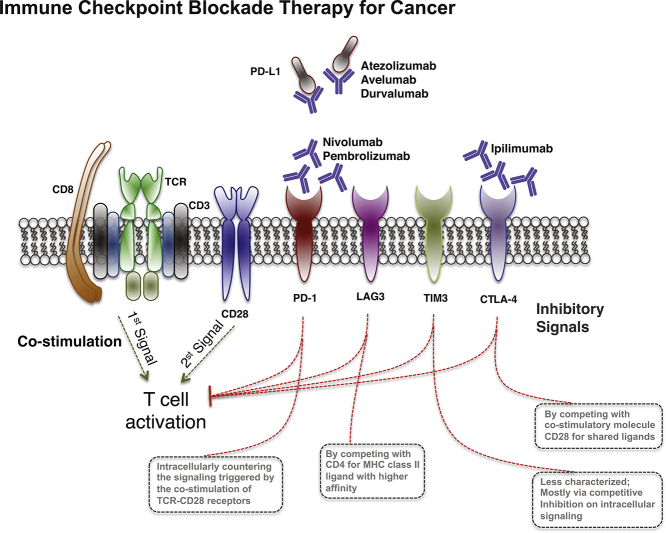

## 1 Introduction

Immune checkpoint inhibitors have emerged as a frontline treatment for multiple malignancies, enabling immunotherapy to join the ranks of surgery, chemotherapy, radiation, and targeted therapy for cancer treatment [1]. Although the first attempt to harness immune system for treating cancer went back to the late nineteenth century [2], the capability of the immune system to recognize and fight cancer remained highly controversial for much of the twentieth century [3,4]. Today, nearly 120 years of basic research in immunology, molecular biology, virology, cell biology, and structural biology have advanced our understanding of the role of the immune system in the surveillance against tumors as well as the strategies exploited by tumor cells to escape such surveillance, ultimately leading to the acceptance of immunotherapy as a promising approach to tackle this dynamic and complex interplay between cancer and immunity [[5], [6], [7], [8]].

To protect the host from any potential threat, the immune system has evolved to impose considerable damage on harmful invaders and efficiently eliminate the most of pathological microbes and toxic substances, but the immune system must accomplish so while sparing healthy cells and maintaining self-tolerance [[9], [10], [11]]. This task is achieved through multiple checkpoints pathways by attenuating immune responses. For instance, cytotoxic T-lymphocyte–associated antigen-4 (CTLA-4) is the best-studied negative regulator of T cells, which modulates T-cell activation by competing with the co-stimulatory molecule CD28 for shared ligands. Among other newly emerged negative regulatory receptors that mediating these inhibitory feedbacks, the programmed cell death protein 1 (PD-1) has been one of the most intensively investigated regulators, due to its indispensable role in fine-tuning T cell's function and maintaining immune system homeostasis. PD-1 acting as a natural brake is capable of eliciting the immune checkpoint response of T cells that commonly associated with periphery tolerance. However, tumor cells take advantage of this checkpoint negative regulation [12] to suppress immunity and evade immune surveillance [13]. The concept of “checkpoint blockade” has been proposed and anti PD-1 pathway agents have been tested to release the immune system's brakes and unleash anti-tumor immune responses in cancer management [[14], [15], [16]].

Successful clinical trials with PD-1/PD-L1 monoclonal antibodies have opened new avenues in cancer immunology, and recent FDA approved PD1 pathway inhibitors are including monoclonal antibody nivolumab (anti-PD1; Bristol-Myers Squibb), pembrolizumab (anti-PD1; Merck), atezolizumab (anti-PD-L1; Genentech/Rothe), avelumab (anti-PD-L1; EMD Serono/Merck&Pfizer) and durvalumab (anti-PD-L1; AstraZeneca). To date, PD-1/PD-L1 checkpoint blockade therapy is part of the standard therapy for multiple malignancies, including melanoma, non-small-cell lung cancer (NSCLC), small cell lung cancer (SCLC), renal cell carcinoma (RCC), classical Hodgkin lymphoma (cHL), head and neck squamous cell carcinoma (HNSCC), colorectal cancer (CRC), hepatocellular carcinoma (HCC), primary mediastinal large B-cell lymphoma (PMLBCL), bladder cancer, Merkel cell carcinoma (MCC), and microsatellite instability high (MSI-H) or DNA mismatch repair deficient (dMMR) adult and pediatric solid tumors, and is intensively being investigated in clinical trials for the treatment of additional malignant conditions.

However, not all patients show complete responses and adverse events (AEs) have been noted, suggesting a better understanding of PD1 pathway mediated immunosuppression is needed to predict patient response and improve treatment efficacy [17]. Here, we first review the molecular mechanisms underlying PD-1 pathway modulated checkpoint response, and then the recent clinical development and commercialization of PD-1 and PD-L1 inhibitors, focusing on the registration trials that leading to FDA-approvals. Following that, we discuss besides cell–intrinsic oncogenic pathways how additional host and environmental factors can have a major impact on immune responses and hence the efficacy of cancer immunotherapeutics. At last, we discuss to use the activity biomarkers for treatment optimization.

## 2 PD-1/PD-L1 Axis in Tumor Evasion

PD-1 is a 50–55-kDa type I transmembrane glycoprotein, whose extracellular domain sharing 21–33% sequence identity with CTLA-4, CD28 and ICOS [18]. PD-1 was discovered in 1992 when Ishida et al. were isolating transiently expressed genes involved in the process of programmed cell death in apoptosis-induced murine T cells [19]. The key physiological function of PD-1 had become clear when knockout mice obtained: PD-1 deficient mice develop different autoimmune diseases depending on their genetic background: C57BL/6-Pdcd1^−/−^ mice develop lupus-like arthritis and glomerulonephritis with predominant IgG3 deposition [20]. BALB/c-Pdcd1^−/−^ mice develop fetal dilated cardiomyopathy (DCM) due to the production of the autoantibody against cardiac troponin I (cTnI) that chronically disturbs the Ca^2+^ homeostasis in cardiomyocytes [21,22]. NOD-Pdcd1^−/−^ mice develop type I diabetes, resulted from extensive destruction of the islets [23]. These observations suggested that PD-1 negatively regulates immune responses and play an essential role in maintaining peripheral self-tolerance, meanwhile the underlying mechanism of PD-1 mediated immunosuppression must have been distinct from that of CTLA-4, as CTL-A deficient mice died of fulminant lymphocytic infiltration of almost all organs [24].

The first evidence of PD-1 signaling pathway implicated in mediating tumor immunity was noted in 2002 [25], in which the overexpression of the ligand of PD-1, PD-L1 [26,27], was found to impair the cytolytic activity of T cells and markedly enhance tumorigensis and tumor invasiveness, and additionally such effects could be reversed by anti-PD-L1 treatment through applying anti-PD-L1 monoclonal antibodies. Further on, accelerated tumor eradication can be observed when employing a variety of approaches of disturbing PD-1 signaling pathway, including antibody blockade of PD-L1, DNA vaccination of the extracellular region of PD-1, tumor-specific T cell clones injection [[28], [29], [30]].

**Fig. 1:**
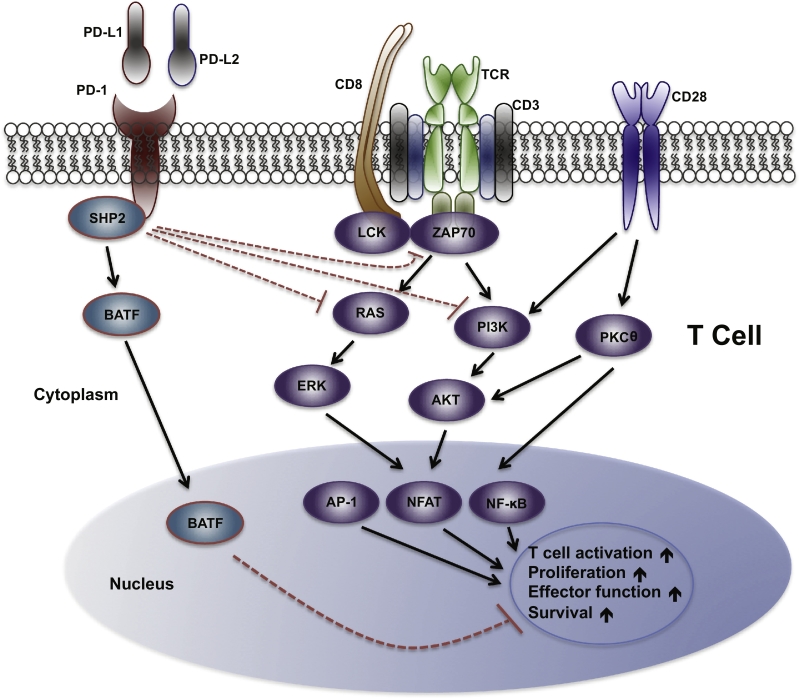
PD-1 signaling pathway in T cells. When engaged with a ligand, PD-1 becomes phosphorylated at its cytoplasmic tyrosine residue, leading to binding of protein tyrosine phosphatases (PTPs), such as SHP2. SHP2 can dephosphorylate kinases and antagonize the positive signals that occur through TCR and CD28 receptors, resulting in inhibition of effort T cell function and T cell exhaustion. Additionally, PD-1 can inhibit T cell functions by increasing the expression of transcription factors such as BATF, which can further counter the signals downstream of TCR and CD28 co-stimulation.

Much of our understanding of PD-1 signaling comes from studies of acutely activated T cells (illustrated in Fig. 1). There are two tyrosine residues located within PD-1 cytoplasmic domain: the membrane-proximal one constituting immune-receptor tyrosine-based inhibitory motif (ITIM) and the other one immune-receptor tyrosine-based switch motif (ITSM) [[31], [32], [33], [34]]. Upon the binding of PD-1 ligands, the tyrosine residue located within ITSM of PD-1 is phosphorylated and recruits the protein tyrosine phosphatases (PTPs), such as SHP2. These PTPs can dephosphorylate various key signaling kinases and counter the positive signaling events triggered by the co-stimulation of TCR-CD28 receptors during the activation of T cells, with a certain level of preference of inhibiting TCR-driven pathways than CD28-mediated pathway. For instance, SHP2 inhibits ZAP70, PI3K-AKT and RAS-ERK rather than PKCθ (shown in the Fig.1). Eventually, PD-1 signaling transduction leads to decreased activation of transcription factors (TFs), such as activator protein 1 (AP1), nuclear factor of activated T cells (NFAT) and NF-κB, to antagonize the positive signals of driving T cell activation, proliferation, effector functions and survival. Moreover, differing from CTLA-4 suppressing T-cell activation early in an immune response and primarily in lymph nodes, PD-1 inhibits T cells later in an immune response primarily in peripheral tissues, allowing PD-1 pathway blockade have a more specific effect on antitumor T cells while exhibiting less toxicity compared to CTLA-4 blockade. (See Tables 1 and 2.)

**Table 1:** Approved therapies based on PD-1/PD-L1 blockade I*.

Pathology		Agent(s) /Approval time	Line of therapy	Control arm	Primary endpoint	Clinical Trial/(n)
Skin cancer	Melanoma	Pembrolizumab/Sep 2014	Previously treated with ipilimumab and/or BRAF inhibitor	/	ORR: 26%	KEYNOTE-001 phase 1 (n = 173)
		Nivolumab/Dec 2014	Previously treated with ipilimumab and/or BRAF inhibitor	ICC	ORR: 31.7% vs. 10.6%	CheckMate-037 phase 3 (n = 370)
		Nivolumab+ipilimumab /Oct 2015	First-line with BRAF WT	Ipilimumab	ORR: 61% vs. 11%	CheckMate-069 phase 2 (n = 140)
		Pembrolizumab/Dec 2015	First-line (regardless of BRAF mutations status)	Ipilimumab	6-month PFS: 47.3%^&^ vs. 46.4%^#^ vs. 26.5% (p < 0.001); 1-year OS: 74.1%* vs. 68.4%^#^ vs. 58.2% (p < 0.005)	KEYNOTE-006 phase 3 (n = 834)
		Nivolumab+ipilimumab /Jan 2016	First-line irrespective of BRAF mutation status	Monotherapy (Ipilimumab or Nivolumab)	Median PFS: 11.5 vs. 6.9 vs. 2.9 months (p < 0.0001); ORR: 50% vs. 40% vs. 14%	CheckMate-067 phase 3 (n = 945)
		Nivolumab/Dec 2017	Stage IIIB/C or IV Adjuvant therapy	Ipilimumab	12-month RFS: 70.5% vs. 60.8%; 16-month RFS: 66.4% vs. 52.7% (p < 0.0001)	CheckMate-238 phase 3 (n = 906)
	MCC	Avelumab/Mar 2017	First-line and beyond	/	ORR: 31.8%	JAVELIN phase 2 (n = 88)
		Pembrolizumab /Dec 2018	First-line and beyond	/	ORR: 56%	KEYNOTE-017 phase 2 (n = 50)
Lung cancer	NSCLC	Nivolumab/Mar 2015	Relapsed/refractoy, squamous	Docetaxel	Median OS: 9.2 vs. 6.0 months (p < 0.001)	CheckMate-017 Phase 3 (n = 272)
		Pembrolizumab /2nd Oct 2015	PD-L1 positive (≥1%) progressing after platinum-based therapy	/	ORR: 41%	CheckMate-057 phase 3 (n = 582)
		Nivolumab /9th Oct 2015	Relapsed/refractory, non-squamous	Docetaxel	Median OS: 12.2 vs. 9.4 months (p = 0.002)	CheckMate-057 phase 3 (n = 582)
		Atezolizumab /18th Oct 2016	Second-line	Docetaxel	POPLAR: OS: 12.6 vs. 9.7 months (p = 0.04); OAK: OS: 13.8 vs. 9.6 months (p = 0.0003)	POPLAR phase 2 (n = 287) & OAK phase 3 (n = 1125)
		Pembrolizumab /24th Oct 2016	First-line; metastatic NSCLC with ≥50% PD-L1 expression with no EGFR or ALK aberrations	ICC	Median PFS: 10.3 vs. 6.0 months (p < 0.001)	KEYNOTE-024 phase 3 (n = 305)
		Pembrolizumab +Pemetrexed & Carboplatin /May 2017	First-line; metastatic nonsquamous NSCLC, irrespective of PD-L1 expression	Placebo	ORR: 55% vs. 29%	KEYNOTE-021phase 2 (n = 123)
		Durvalumab /Feb 2018	Stage III, unresectable, and no progression after chemoradiation	Placebo	Median PFS: 16.8 vs 5.6 months (p < 0.001)	PACIFIC phase 3 (n = 713)
		Pembrolizumab +Pemetrexed & platinum /August 2018	First-line; metastatic nonsquamous NSCLC, with no EGFR or ALK aberrations	Placebo	ORR: 62.9% vs. 49.4% median PSF: 8.8 vs. 4.9 months (p < 0.001)	KEYNOTE-189 phase 3 (n = 616)
	SCLC	Nivolumab/Aug 2018	Recurrent, after platinum-based treatment	/	ORR: 11.9%; median DOR: 17.9 months	CheckMate-032 phase ½ (n = 109)
Blood cancer	cHL	Nivolumab/May 2016	Previously treated with ASCT or brentuximab	/	ORR: 65%; median DOR: 8.7 months	CheckMate-039 phase 1 & CheckMate-205 phase 2 (n = 95)
		Pembrolizumab/Mar 2017	Relapsed after ≥3 lines of therapy or refractory	/	ORR: 69%; median DOR: 11.1 months	KEYNOTE-087 phase 2 (n = 210)
	PMBCL	Pembrolizumab/Jun 2018	Relapsed after ≥2 lines of therapy	/	ORR: 45%	KEYNOTE-170 phase 2 (n = 53)

*With relatively high response rate; ^&^Pembrolizumab 10 mg/kg every 2 weeks; ^#^Pembrolizumab 10 mg/kg every 3 weeks; ICC: Investigator's choice of chemotherapy; ORR: Overall response rate; OS: Overall survival; DOR: Duration of response; PFS: Progression-free survival; NSCLC: Non-small cell lung cancer; cHL: classical Hodgkin lymphoma; SCLC: Small cell lung cancer; PMBCL: Primary mediastinal large B-cell lymphoma; MCC: Merkel cell carcinoma; ASCT: autologous hematopoietic stem cell transplantation.

**Table 2:** Approved therapies based on PD-1/PD-L1 blockade II*.

Pathology		Agent(s)/Approval time	Line of therapy	Control arm	Primary endpoint	Clinical trial/(n)
Renal cancer	UC	Atezolizumab/May 2016	Recurrent, after platinum-based treatment	/	ORR: 14.8%	IMVigor 210 phase 2 (n = 310)
		Nivolumab/Feb 2017	Recurrent, after platinum-based treatment	/	ORR: 19.6%; median DOR: 10.3 months	CheckMate-275 phase 2 (n = 270)
		Atezolizumab/Apr 2017	First-line cisplatin ineligible	/	ORR: 23.5%	IMVigor 210 phase 2 (n = 119)
		Durvalumab /1st May 2017	Recurrent, after platinum-based treatment	/	ORR: 17.0%	Study 1108 phase 2 (n = 191)
		Avelumab/9th May 2017	Recurrent, after platinum-based treatment	/	ORR: 13.3%	JAVELIN Solid Tumor phase 1 (n = 242)
		Pembrolizumab /18th May 2017	First-line cisplatin- ineligible	/	ORR: 29%	KEYNOTE-052 phase 2 (n = 370)
		Pembrolizumab /18th May 2017	Recurrent, after platinum-based treatment	ICC	OS: 10.3 vs. 7.4 months (p = 0.002)	KEYNOTE-045 phase 3 (n = 542)
	RCC	Nivolumab/Nov 2015	Second-line	Everolimus	Median OS: 25 vs.19.6 months (p = 0.002); ORR: 21.5% vs. 3.9%	CheckMate-025 phase 3 (n = 821)
		Nivolumab+ipilimumab /Apr 2018	First-line	Sunitinib	ORR: 42% vs. 27% (p < 0.001) Median PFS: 11.6 vs 8.4 months (p > 0.05)	CheckMate-214 phase 3 (n = 847)
Gastrointestinal cancers	HCC	Nivolumab /Sep 2017	Second-line after sorafenib	/	ORR: 18.2%	CheckMate-040 phase ½ (n = 154)
		Pembrolizumab /Dec 2018	Second-line after sorafenib	/	ORR: 17%	KEYNOTE-224 phase 2 (n = 104)
	GC	Pembrolizumab /Sep 2017	PD-L1 ≥ 1% and progression on ≥2 lines of chemotherapy	/	ORR: 13.3%	KEYNOTE-059 phase 2 (n = 143)
	MSI-H and dMMR ^$^	Pembrolizumab /May 2017	Treatment-refractory to all standard therapies	/	ORR: 26.2%	KEYNOTE-164 phase 2 (n = 63)
		Nivolumab/Aug 2017	Treatment-refractory to all standard therapies	/	ORR: 31.1%	CheckMate-142 phase 2 (n = 74)
		Nivolumab+ipilimumab /Jul 2018	Treatment-refractory to all standard therapies	/	ORR: 49%	CheckMate-142 phase 2 (n = 119)
Other solid tumors	HNSCC	Pembrolizumab/Aug 2016	Recurrent, after platinum-based treatment and PD-L1 ≥ 1%	/	ORR: 16%	KEYNOTE-012 phase 1b (n = 60)
		Nivolumab/Nov 2016	Recurrent, after platinum-based treatment	ICC	Median OS: 7.5 vs. 5.1 months (p = 0.01); ORR: 13.3% vs. 5.8%	CheckMate-141 phase 3 (n = 361)
	CC	Pembrolizumab/Jun 2018	Refractory or metastatic, with PD-L1 ≥ 1%	/	ORR: 14.3%; median DOR: not reached	KEYNOTE-158 phase 2 (n = 77)

*Usually with intermediate response rate; ^$^MSI-H or dMMR unresectable or metastatic solid tumors, including colorectal, endometrial and other gastrointestinal cancers; ICC: Investigator's choice of chemotherapy; ORR: Overall response rate; OS: Overall survival; DOR: Duration of response; PFS: Progression-free survival; RCC: Renal cell carcinoma; HNSCC: Head and neck squamous cell carcinoma; UC: Urothelial carcinoma; CRC: Colorectal cancer; HCC: Hepatocellular carcinoma; MSI-H: Microsatellite instability-high; dMMR: DNA mismatch repair deficiency; GC: Gastric cancer; CC: Cervical cancer.

The ligands for PD-1 are PD-L1 (B7—1H) [26] and PD-L2 (B7-DC) [35,36]. PD-L1 is widely expressed by many different cell types and is found on both hematopoietic cells (including T cells, B cells, dendritic cells and macrophages) and on non-hematopoietic cells (including vascular endothelium, pancreatic islet cells, placental syncytiotrophoblasts and keratinocytes). PD-L1 expression in the normal tissues is a major mechanism of physiologic peripheral immune tolerance to control tissue autoimmune responses after sustained inflammatory response to tissue damage. However, tumor cells could also exploit PD-L1 as a molecular “shield” to attenuate T cell-mediated cytotoxicity to evade immune surveillance. By contrast, the constitutive basal expression of PD-L2 is very low and with much more restricted expression pattern, predominantly on DCs, macrophages and B cell populations, making it so far a less interested therapeutic target in cancer immunotherapy [17,37]. Over the years, PD-1/PD-L1 axis has become the paradigm for understanding a variety of physiological roles of inhibitory receptors in the immune system and how the signals through the PD-1 pathway contributing to fine-tuning of T cell fate and functions [17].

## 3 Clinical Application of PD-1 Blockade

Conventional therapies usually target a particular molecule in the tumor cells, in which most tumor responses last until the cancer develops a way to bypass the blocked pathway, whereas PD-1 blockade releasing negative regulators of immune checkpoints is applicable to a wide range of malignancies as well as provides long-lasting responses. The highest antitumor activities of single-agent PD-1 blockade therapy have been observed in carcinogen-induced cancers or malignancies driven by viral infections, such as classic Hodgkin's lymphoma (cHL), the virally induced Merkel cell carcinoma of the skin, microsatellite-instability high (MSI-H) cancers and desmoplastic melanoma, leading to response rates that can reach 50 to 80% [38]. A second group of cancers with relatively high response rates are cancer with a relative high immunogenicity, such as melanoma and NSCLC, RCC and HCC, with objective response rate ranging from 20% to 40%.

Since the approval of pembrolizumab for the treatment of advanced melanoma in September 2014, to date, at least 500 clinical studies with PD-1 signal inhibitors have been conducted with nine types of antibodies from eight pharmaceutical companies on at least 20 types of solid and hematological malignant tumors [38]. According to a clinical trials database managed by the U.S. National Institutes of Health (https://clinicaltrials.gov). The total number of subjects worldwide is >20,000. Here, we review the registration trials that have successfully led to FDA-approval and the commercialization of PD-1 and PD-L1 inhibitors.

### 3.1 Nivolumab (Opdivo, Bristol-Myers Squibb)

Humanized monoclonal IgG4 anti-PD-1 antibody approved for the treatment of unresectable, metastatic or completely resected melanoma, metastatic NSCLC, urothelial carcinoma (UC), advanced RCC, HNSCC, cHL, CRC and HCC. Nivolumab acts by binding to the PD-1 receptor and blocking of its interaction with both PD-L1 and PD-L2, thereby releasing PD-1 pathway mediated immune suppression to against tumor cells.

#### 3.1.1 Melanoma

In December 2014, nivolumab was first approved as second-line treatment of unresectable or metastatic melanoma based on the results of an open-label phase 3 clinical trial CheckMate-037 [39]. The overall response rate (ORR) of Nivolumab was 31.7% (95% CI: 23.5–40.8), contrasting to 10.6% (95% CI: 3.5–23.1) with patients treated with chemotherapy. Nivolumab led to a greater proportion of patients achieving an objective response and fewer toxic effects than with alternative available chemotherapy regimens for patients with advanced melanoma that has progressed after ipilimumab, or ipilimumab and a BRAF inhibitor if they were BRAF V600 mutation-positive. Three years later, in December 2017, nivolumab was granted FDA approval as adjuvant therapy for patients with completely resected melanoma with lymph node involvement or metastatic disease, based on the results of the randomized, double blind, phase 3 trial CheckMate-238 [40]. In this study, the 12-month rate of recurrence-free survival (RFS) was 70.5% (95% CI: 66.1–74.5) in the nivolumab group and 60.8% (95% CI: 56.0–65.2) in the ipilimumab group, demonstrating 10% higher efficacy of nivolumab versus ipilimumab in adjuvant therapy in terms of relapse-free survival.

The combination of nivolumab and ipilimumab was approved as first-line treatment for BRAFV600-wildtype unresectable or metastatic melanoma in October 2015 based on results from CheckMate-069 [41]. This randomized, double-blinded phase 2 trial, compared nivolumab 1 mg/kg in combination with ipilimumab 3 mg/kg (every 3 weeks x4 cycles then nivolumab alone every 2 weeks) against ipilimumab 3 mg/kg monotherapy (every 3 weeks) as first-line treatment. Objective response occurred in 61% of patients with BRAFV600-wild-type tumors in the combination group compared with 11% of patients in the monotherapy group, and complete responses were obtained in 22% of patient in the combination group and no patients in the ipilimumab-monotherapy group.

In January 2016, nivolumab and ipilimumab combination therapy received an expanded approval for unresectable or metastatic melanoma irrespective of BRAFV600 mutation status based on results of the CheckMate-067 trial [42,43]. In this phase 3 trial, patients with untreated, unresectable or metastatic melanoma were randomized to receive nivolumab 3 mg/kg every 2 weeks, nivolumab 1 mg/kg and ipilimumab 3 mg/kg every 3 weeks for 4 doses followed by nivolumab 3 mg/kg every 2 weeks, or ipilimumab 3 mg/kg. The median progression-free survival (PFS) was 11.5 months (95% CI: 8.9–16.7) with nivolumab plus ipilimumab, as compared with 2.9 months (95% CI: 2.8–3.4) with ipilimumab, and 6.9 months (95% CI: 4.3–9.5) with nivolumab. Longer overall survival (OS) was demonstrated with nivolumab and combination therapy compared with ipilimumab alone across BRAFV600 status.

#### 3.1.2 Lung Cancer

Nivolumab was approved as treatment for metastatic squamous NSCLC in March 2015 based on the phase 3 trail CheckMate-017 [44]. Patients were randomized to receive nivolumab 3 mg/kg every 2 weeks or docetaxel 75 mg/m^2^ every 3 weeks, and the median OS was 9.2 months (95% CI: 7.3–13.3) with nivolumab versus 6.0 months (95% CI: 5.1–7.3) with docetaxel. The risk of death was 41% lower with nivolumab than with docetaxel (P < 0.001). At 1 year, the OS rate was 42% with nivolumab versus 24% with docetaxel. The response rate was 20% with nivolumab versus 9% with docetaxel (P = 0.008) regardless the expression of PD-L1. Later on, FDA expanded the approval of nivolumab to metastatic nonsquamous NSCLC in October in the same year based on the CheckMate-057 trial [45]. This phase 3 trial enrolled the patients who had progressed during or after platinum-based doublet chemotherapy to receive nivolumab 3 mg/kg every 2 weeks or docetaxel 75 mg/m^2^ every 3 weeks. The primary endpoint was OS, which were 12.2 months with nivolumab (95% CI: 9.7–15.0) and 9.4 months with docetaxel (95% CI: 8.1–10.7).

Further on, in August 2018 Nivolumab received approval from FDA as the first and only immuno-oncology treatment option for patients with metastatic small cell lung cancer (SCLC) whose cancer has progressed after platinum-based chemotherapy and at least one other line of therapy, based on the ongoing Phase 1/2 trail CheckMate-032 (https://clinicaltrials.gov/ct2/show/NCT01928394), in which patients received 3 mg/kg of nivolumab every 2 weeks. The ORR is 11.9% (95% CI: 6.5–19.5) to treatment based on assessment by a Blinded Independent Central Review (BICR), regardless of PD-L1 expression, 11% patients had a partial response and one patient had a complete response (0.9%). Among these responders, the median duration overall response (DOR) was 17.9 months (95% CI: 7.9–42.1).

#### 3.1.3 Renal Cancer

Nivolumab was approved for metastatic RCC in Nov 2015 based on the efficacy and safety results of an open-label randomized phase 3 trial CheckMate-025 [46]. Survival benefit was demonstrated in favor of nivolumab when compared to everolimus in patients who had received one or two prior antiangiogenic therapies. The median OS was 25.0 months (95% CI: 21.8-not estimable) with nivolumab and 19.6 months (95% CI: 17.6–23.1) with everolimus. The ORR was greater with nivolumab than with everolimus (21.5% vs. 3.9%; P < .001). Survival benefit was observed in every subgroup receiving nivolumab over everolimus irrespective of PD-L1 expression.

In April 2018, nivolumab and ipilimumab combination therapy received an approval as first-line treatment for patients with intermediate-and poor risk advanced RCC based on results of the CheckMate 214 trial [47]. Patients were randomly assigned to receive either nivolumab 3 mg/kg plus ipilimumab 1 mg/kg every 3 weeks for four doses, followed by nivolumab 3 mg/kg every 2 weeks, or sunitinib 50 mg orally once daily for 4 weeks (6-week cycle). At a median follow-up of 25.2 months in intermediate- and poor-risk patients, the 18-month overall survival rate was 75% (95% CI: 70–78) with nivolumab plus ipilimumab and 60% (95% CI: 55–65) with sunitinib. The objective response rate was 42% versus 27% (P < 0.001), and the complete response rate was 9% versus 1%. The median progression-free survival was 11.6 months and 8.4 months, respectively. Taken together, OS and ORR were significantly higher with nivolumab plus ipilimumab than with sunitinib among intermediate- and poor-risk patients with previously untreated advanced renal-cell carcinoma.

#### 3.1.4 Hodgkin Lymphoma

In May 2016, nivolumab received the first approval for the treatment of patients with classical Hodgkin lymphoma (cHL) who have relapsed or progressed after autologous hematopoietic stem cell transplantation and post-transplantation brentuximab vedotin (BV) through an expedited review process, based on two single-arm, phase 2, multicenter trials (CheckMate-205 and CheckMate-039 [48,49]). This was the first FDA application and approval for a PD-1 inhibitor as treatment for hematological malignancies and it was based on the improved response rate and duration demonstrated in these two single-arm phase 2 studies. Nivolumab delivered a high response rate with ORR of 65% (CI 95%: 55–75), including 7% complete response (CI 95%: 3–15) and 58% partial response (CI 95%: 47–68). Among responders, the duration of response was maintained over time for a median of 8.7 months.

#### 3.1.5 HNSCC

In November 2016, nivolumab became the first immunotherapy approved by the FDA in the treatment for recurrent or metastatic HNSCC with disease progression on or after platinum-based therapy, based on the results from phase 3 randomized trial CheckMate-141 [50]. Nivolumab demonstrated statistically significant and clinically meaningful superior OS versus the comparator arm (investigator's choice of methotrexate, docetaxel or cetuximab), with a 30% reduction in the risk of death. The median OS was 7.5 months (95% CI: 5.5–9.1) for nivolumab compared to 5.1 months (95% CI: 4.0–6.0) for investigator's choice.

#### 3.1.6 Urothelial Carcinoma

In February 2017, The FDA approved nivolumab for locally advanced or metastatic UC following the results from CheckMate-275 [51]. This phase 2 study enrolled patients who had experienced progression or recurrence after ≥1 platinum-based chemotherapy regimen to receive nivolumab 3 mg/kg every 2 weeks. ORR was higher in patients with high PD-L1 expression (28.4% for PD-L1 ≥ 5%, 23.8% for PD-L1 ≥ 1%, and 16.1% for PD-L1 < 1%) whereas OS rate was also higher for tumors with PD-L1 expression of ≥1% versus PD-L1 < 1%.

#### 3.1.7 Colorectal Cancer

In August 2017, nivolumab was approved in DNA mismatch repair deficiency or a microsatellite instability-high (dMMR/MSI-H) metastatic colorectal cancer refractory to fluoropyrimidine, oxaliplatin, and irinotecan, based on the results of the CheckMate-142 trial [52]. In this phase 2 trial, patients received nivolumab 3 mg/kg every 2 weeks as a single agent and were stratified by PD-L1 < 1% and PD-L1 ≥ 1%. The ORR was 31.1% (95% CI: 20.8–42.9) irrespective of the PD-L1 expression levels. In July 2018, nivolumab and low-dose ipilimumab combination therapy received the approval for the same type of colorectal cancer, following the results of CheckMate-142 trial which evaluated the combination of nivolumab 3 mg/kg plus low-dose ipilimumab 1 mg/kg in patients with MSI-H or dMMR metastatic CRC previously treated with chemotherapy (https://clinicaltrials.gov/ct2/show/NCT02060188?term=NCT02060188). Approval for this indication has been granted under accelerated approval based on ORR and duration overall response (DOR). The ORR was 49% (95% CI: 39–58) with 4.2% complete response and 45% partial response. Among these responders, the median DOR was not reached (range: 1.9–23.2+ months) and 83% of those patients had responses of six months or longer, and 19% had responses of 12 months or longer.

#### 3.1.8 Hepatocellular Carcinoma

In September 2017, Nivolumab was approved for the treatment of patients with advanced HCC who have been previously treated with sorafenib during the phase 1/2 escalation and expansion CheckMate-040 trial [53]. The ORR reached 18.2% (95% CI: 12.4–25.2) regardless of either the expression of PD-L1 or the presence of viral hepatitis, with complete response rate 3.2%. Among responders, responses ranged from 3.2 to 38.2+ months; 91% of those patients had responses of six months or longer and 55% had responses of 12 months or longer.

### 3.2 Pembrolizumab (Keytruda, Merck & Co/MSD)

Humanized mAb targeting PD-1. It is approved for use against metastatic or unresectable melanoma, metastatic NSCLC, advanced UC, recurrent or metastatic HNSCC, cHL, any unresectable or metastatic solid tumor with MSI-H/dMMR state, advanced cervical cancer, advanced or metastatic gastric or gastroesophageal junction adenocarcinoma and refractory or relapsed primary mediastinal large B-Cell lymphoma (PMBCL). Its mechanism of action is similar to that of Nivolumab in that it disrupts the binding of PD-1 with its immune-suppressing ligands, inhibiting the suppression of T-cell response and leading to effective immune response.

#### 3.2.1 Melanoma

In September 2014, pembrolizumab was approved as second line treatment for patients with advanced or unresectable melanoma, becoming the first PD-1 inhibitor to receive approval for such indication. The role of pembrolizumab in advanced melanoma was explored in the Phase 1 KEYNOTE-001 trial [54], in which patients with advanced or unresectable melanoma who had previously failed treatment with ipilimumab and a BRAF inhibitor (if BRAFV600-mutated) were treated with pembrolizumab. The primary study endpoint was ORR per RECIST 1.1. The ORR was 26% in both the pembrolizumab 2 mg/kg and 10 mg/kg groups. In an update of KEYNOTE-001, it was shown that the PFS at 6 months was 45%, median OS was 25.9 months, and ORR 34% in ipilimumab-treated and 54% in ipilimumab-naïve patients.

In December 2015, pembrolizumab was approved for expanded indication and became the first-line treatment of patients with unresectable or metastatic melanoma based on the phase 3 KEYNOTE-006 trials [55,56]. In the study supporting the first-line approval, patients given pembrolizumab 10 mg/kg every two weeks demonstrated a 37% reduction in the risk of death and those given pembrolizumab 10 mg/kg every 2 or 3 weeks demonstrated a 42% reduction in the risk of disease progression or death as compared to ipilimumab. Median PFS was 5.5 months (95% CI: 3.4–6.9), 4.1 months (95% CI: 2.9–6.9), and 2.8 months (95% CI: 2.8–2.9) with pembrolizumab 10 mg/kg every 2 weeks, pembrolizumab 10 mg/kg every 3 weeks and ipilimumab, respectively. The ORR was 34% (95% CI: 28–40) with pembrolizumab 10 mg/kg every 2 weeks and 33% (95% CI: 27–39) with pembrolizumab 10 mg/kg every 3 weeks, as compared with 12% (95% CI: 8–16) with ipilimumab.

#### 3.2.2 Lung Cancer

In October 2015, pembrolizumab was approved for treatment of previously treated advanced or metastatic PD-L1-positive (at least 1%) NSCLC, based on the results of the randomized phase 1b trial KEYNOTE-001 [57], in which patients received pembrolizumab 2 or 10 mg/kg every 3 weeks or 10 mg/kg every 2 weeks. The ORR for those receiving pembrolizumab 2 mg/kg was 28% (95% CI: 12.1–49.4%) as compared to 40% (95% CI: 22.4–61.2) and 41% (95% CI: 24.7–59.3%) in patients receiving pembrolizumab 10 mg/kg every 2 weeks and every 3 weeks. Pembrolizumab was approved under the agency's accelerated approval program, allowing earlier patient access to new drug while the company conducts confirmatory clinical trials. Following KEYNOTE-001, KEYNOTE-010 was a phase 2/3 clinical trial [58], in which patients with EGFR mutation or ALK rearrangement following failure of platinum-based chemotherapy or a tyrosine kinase inhibitor received either pembrolizumab (at 2 mg/kg or 10 mg/kg) or docetaxel (75 mg/m^2^ every 3 weeks) for treatment. The OS was significantly longer for pembrolizumab 2 mg/kg versus docetaxel (p = 0.0008) and for pembrolizumab 10 mg/kg versus docetaxel (p < 0.0001). Among patients with at least 50% of tumor cells expressing PD-L1, overall survival was significantly longer with pembrolizumab 2 mg/kg than with docetaxel (14.9 months vs 8.2 months; p = 0.0002) and with pembrolizumab 10 mg/kg than with docetaxel (17.3 months vs 8.2 months; p < 0.0001). Likewise, for this patient population, PFS was significantly longer with pembrolizumab 2 mg/kg than with docetaxel (5.0 months vs 4.1 months; p = 0.0001) and with pembrolizumab 10 mg/kg than with docetaxel (5.2 months vs 4.1 months; p < 0.0001).

In October 2016, FDA has approved pembrolizumab for the first-line treatment of patients with metastatic NSCLC whose tumors have high PD-L1 expression (≥50% PD-L1 expression) with no EGFR or ALK genomic tumor aberrations based on the study of KEYNOTE-024 [59]. KEYNOTE-024 was a randomized, open-label, phase 3 study evaluating pembrolizumab monotherapy compared to standard of care platinum-containing chemotherapy for the treatment of patients with both squamous (18%) and non-squamous (82%) metastatic NSCLC. The study randomized patients to receive pembrolizumab (200 mg every 3 weeks) or investigator-choice platinum-based chemotherapy. Pembrolizumab reduced the risk of progression or death by 50% compared to chemotherapy (p < 0.001) and the risk of death 40% compared to chemotherapy (p = 0.005), indicating pembrolizumab was superior compared to chemotherapy for both the primary endpoint of PFS and the secondary endpoint of OS.

In May 2017, pembrolizumab was approved in combination with pemetrexed and carboplatin for the first-line treatment of metastatic nonsquamous NSCLC, irrespective of PD-L1 expression, based on the study of KEYNOTE-021 [60]. In the phase 2 open-label trial, 55% patients in the pembrolizumab plus chemotherapy group achieved an objective response compared with 29% patients in the chemotherapy alone group (p = 0.0016). In August 2018, FDA has approved an expanded label for pembrolizumab in combination with pemetrexed and platinum chemotherapy for the first-line treatment of patients with metastatic nonsquamous NSCLC, with no EGFR or ALK genomic tumor aberrations, based on results of the KEYNOTE-189 trial [61]. OS was 69.2% (95% CI: 64.1–73.8) in the pembrolizumab-combination group versus 49.4% (95% CI: 42.1–56.2) in the placebo-combination group (p < 0.001), regardless of PD-L1 tumor expression status. Median PFS was 8.8 months (95% CI: 7.6–9.2) in the pembrolizumab-combination group and 4.9 months (95% CI: 4.7–5.5) in the placebo-combination group (p < 0.001). Pembrolizumab in combination with pemetrexed and platinum chemotherapy demonstrated a statistically significant and clinically meaningful improvement in OS and PFS, reducing the risk of death by half compared to chemotherapy alone.

#### 3.2.3 HNSCC

In August 2016, pembrolizumab was approved for the treatment of patients with recurrent or metastatic HNSCC with disease progression on or after platinum-containing chemotherapy, based on the study of phase 1b clinical trial KEYNOTE-012 [62]. This open-label, multicenter clinical trial examined safety and efficacy of single agent pembrolizumab in the management of the patients with any level of PD-L1 expression (at least 1% of tumor cells or stroma that were PD-L1-positive by immunohistochemistry) receiving pembrolizumab 10 mg/kg every 2 weeks. Safety of the drug was acceptable and ORR was 18% (95% CI: 8–32) in all patients and was 25% (95% CI: 7–52) in HPV-positive patients and 14% (95% CI: 4–32) in HPV-negative patients.

#### 3.2.4 Hodgkin Lymphoma

In March 2017, pembrolizumab was approved for the treatment of adult and pediatric patients with refractory cHL, or who have relapsed after three or more prior lines of therapy, based on the phase 2 trial KEYNOTE-087 [63]. In this single-arm trial, three cohorts of patients with relapsed or refractory classical HL, defined on the basis of lymphoma progression after (1) autologous stem cell transplantation (ASCT) and subsequent medication of brentuximab vedotin (BV); (2) salvage chemotherapy and BV, and thus, ineligible for ASCT because of chemo-resistant disease; and (3) ASCT, but without BV after transplantation, and all adult patients received pembrolizumab 200 mg once every 3 weeks and pediatric patients at a dose of 2 mg/kg (up to a maximum of 200 mg). In all three groups, the ORR was high (73.9%, 64.2% and 70.0% respectively) and toxicity was manageable as well. These results led to the only approval of pembrolizumab for a hematological malignancy.

#### 3.2.5 Urothelial Cancer

In May 2017, pembrolizumab received 2 FDA approvals: for the firs-line setting in patients with locally advanced or metastatic UC who are cisplatin-ineligible based on the single-arm, phase 2 clinical trial KEYNOTE-052 [64], and for the second-line setting in patients with locally advanced or metastatic UC who have disease progression after platinum-containing chemotherapy based on the phase 3 KEYNOTE-045 trial [65]. In KEYNOTE-052 trial, enrolled patients received intravenous pembrolizumab 200 mg every 3 weeks. The ORR reached 29% (95% CI: 24–34) and PD-L1-expression cutoff of 10% was associated with a higher frequency of response to pembrolizumab (38%, 95% CI: 29–48). In the phase 3, international KEYNOTE-045 trial, patients with advanced UC showing ≥10% PD-L1 expression who recurred or progressed after platinum-based chemotherapy were randomized to receive pembrolizumab 200 mg every 3 weeks or the investigator's choice of chemotherapy with paclitaxel, docetaxel or vinflunine. The median OS in the total population was 10.3 months (95% CI: 8.0–11.8) in the pembrolizumab group, as compared with 7.4 months (95% CI: 6.1–8.3) in the chemotherapy group (P = 0.002). The median OS among patients who had a tumor PD-L1 combined positive score of 10% or more was 8.0 months (95% CI: 5.0–12.3) in the pembrolizumab group, as compared with 5.2 months (95% CI: 4.0–7.4) in the chemotherapy group (P = 0.005). Pembrolizumab was associated with significantly longer OS (by approximately 3 months) as second-line therapy for platinum-refractory advanced UC.

#### 3.2.6 Microsatellite Instability High (MSI-H) or Mismatch Repair Deficient (d-MMR) Cancers

In May 2017, FDA approved the use of pembrolizumab for patients with unresectable or metastatic solid tumors bearing either of these two biomarkers referred to as microsatellite instability-high (MSI-H) or mismatch repair deficient (d-MMR). A number of single-arm trials have shown impressive response rates with pembrolizumab in patients with MSI-H and d-MMR solid tumors, including colorectal, endometrial and other gastrointestinal cancers [[66], [67], [68], [69]]. The review of pembrolizumab for this indication was based on the percentage of patients who experienced complete or partial shrinkage of their tumors and durability of response. 39.6% of patients who received pembrolizumab in the trials had a complete or partial response. For 78% of those patients, the response lasted for six months or more. The ORR is reported as 42.9% (95% CI: 21.8–66.0%) for non-colorectal MSI-H cancers according to ongoing trial KEYNOTE-158 (https://clinicaltrials.gov/ct2/show/NCT02628067) and 26.2% (95% CI: 15.8–39.1%) for colorectal MSI-H cancers based on KEYNOTE-164 (https://clinicaltrials.gov/ct2/show/NCT02460198).

#### 3.2.7 Gastric Cancer

In September 2017, pembrolizumab was granted its approval for the treatment of patients with recurrent locally advanced or metastatic gastric or gastroesophageal junction (GEJ) adenocarcinoma whose tumors express PD-L1. This was based on the results of the multicenter, non-randomized, open-label, KEYNOTE-059 trial which enrolled patients with gastric or GEJ adenocarcinoma who progressed on at least two prior systemic treatments for advanced disease (http://clinicaltrials.gov/show/NCT02335411). This indication is approved under the FDA's accelerated approval regulations based on tumor response rate and durability of response. By the time of approval, the ORR reached 13.3% (95% CI: 8.2–20.0) with a complete response rate of 1.4% and a partial response rate of 11.9%. Among the responding patients, the duration of response ranged from 2.8+ to 19.4+ months, with 58% patients having responses of six months or longer and 26% patients having responses of 12 months or longer. According to latest released report of KEYNOTE-059 trial, the ORR and median ROD were 15.5% (95% CI: 10.1–22.4) and 16.3 (1.6+ to 17.3+) months [70].

#### 3.2.8 Cervical Cancer

In June 2018, pembrolizumab was approved for the treatment of patients with recurrent or metastatic CC with disease progression on or after chemotherapy whose tumors express PD-L1 ≥ 1%. In study KEYNOTE-158, a multi-center, non-randomized, open-label, multi-cohort trial, patients were treated with pembrolizumab intravenously at a dose of 200 mg every 3 weeks until unacceptable toxicity or documented disease progression. The ORR reached 14.3% (95% CI: 7.4–24.1), with 2.6% complete response percent and 11.7% partial response. Among the responding patients, median DOR was not yet reached (range, 4.1 to 18.6+ months) and 91% experienced DOR of six months or longer. The median follow-up time was 11.7 months (range, 0.6 to 22.7 months). Based on tumor response rate and DOR, FDA's accelerated approval was given.

#### 3.2.9 PMBCL

In June 2018, pembrolizumab was approved for the treatment of adult and pediatric patients with refractory primary mediastinal large B-cell lymphoma (PMBCL), or who have relapsed after two or more prior lines of therapy. This indication is approved under the FDA's accelerated approval regulations based on data from a multicenter, open-label, single-arm trial KEYNOTE-170 (https://clinicaltrials.gov/ct2/show/NCT02576990). Patients received pembrolizumab 200 mg every 3 weeks until unacceptable toxicity or documented disease progression, or for up to 24 months for patients who did not progress. The ORR was 45% (95% CI: 32–60), with a complete response rate of 11% and a partial response rate of 34%. For the patients who responded, the median time to first objective response (complete or partial response) was 2.8 months (range, 2.1 to 8.5 months).

#### 3.2.10 HCC

In November 2018, FDA granted accelerated approval to pembrolizumab for patients with HCC who have disease progression on or after sorafenib or were intolerant to sorafenib, based on a single-arm, multicenter phase 2 trial KEYNOTE-224. The ORR was 17% (95% CI: 11–26), with one complete response and 17 partial responses out of 104 participants. Among the responding patients, response durations ranged from 3.1 to 16.7 months; 89% of responders had response durations of six months or longer, and 56% had response durations of 12 months or longer.

#### 3.2.11 MCC

In December 2018, FDA granted accelerated approval to pembrolizumab for adult and pediatric patients with recurrent locally advanced or metastatic MCC, based on a multicenter, non-randomized, open-label phase 2 trial KEYNOTE-017. The ORR was 56% (95% CI: 41–70) with a complete response rate of 24%. The median response duration was not reached. Among the patients with responses, 96% had response durations of >6 months and 54% had response durations of >12 months.

### 3.3 Atezolizumab (Tecentriq, Genetech/Roche)

IgG1 mAb against PD-L1, which has been approved to treat metastatic NSCLC and locally advanced or metastatic UC in first and second line setting. Atezolizumab binds to the ligand PD-L1 on tumor cells resulting in a blockade of the PD-L1 binding to its inhibitory receptor PD-1.

#### 3.3.1 Urothelial Cancer

In May 2016, atezolizumab became the first PD-L1 inhibitor approved for locally advanced and metastatic UC whose disease had progressed after previous platinum-based chemotherapy based on results of IMVigor 210 [71]. Patients received treatment with intravenous atezolizumab (1200 mg, given every 3 weeks). PD-L1 expression on tumor-infiltrating immune cells was assessed prospectively by immunohistochemistry. The results of the multicenter phase 2 study IMVigor 210 showed 14.8% (95% CI: 11–20) of participants experienced at least a partial shrinkage of their tumors, an effect that lasted from >2.1 to >13.8 months at the time of the response analysis. Increased levels of PD-L1 expression on immune cells were associated with increased response with PD-L1 ≥ 5% showing a 26% response (95% CI: 18–36) and PD-L1 ≥ 1% showing 18% response (95% CI: 13–24) (compared to 9.5% of participants who were classified as “negative” for PD-L1 expression).

In April 2017, accelerated approval of atezolizumab in the first-line treatment of people with locally advanced or metastatic UC who are not eligible for cisplatin chemotherapy, based on a separate cohort of the IMVigor 210 trial [72]. Patients were given 1200 mg intravenous atezolizumab every 21 days until progression. The ORR was 23.5% (95% CI: 16.2–32.2) with the complete response rate was 6.7%. Responses occurred across all PD-L1 and poor prognostic factor subgroups. Median PFS was 2.7 months (95% CI: 2.1 to 4.2). Median overall survival was 15.9 months (10.4 to not estimable). Atezolizumab showed encouraging durable response rates, survival, and tolerability, supporting its therapeutic use in untreated metastatic UC.

#### 3.3.2 NSCLC

In October 2016, atezolizumab was approved for the patients with metastatic NSCLC who have disease progression during or following platinum-containing chemotherapy, and have progressed on an appropriate FDA-approved targeted therapy if their tumor has EGFR or ALK gene abnormalities, based on the results of the phase 2 POPLAR [73] and phase 3 OAK trials [74]. In single-arm, two-cohort, POPLAR trial, patients received treatment with intravenous atezolizumab (1200 mg, given every 3 weeks). The ORR reached 26% (95% CI: 18–36) with PD-L1 ≥ 5%, 18% (95% CI: 13–24) with PD-L1 ≥ 1%, and 15% (95% CI: 11–19) overall in all patients, significantly higher than the historical control rate of 10%. The primary endpoint was OS and at a minimum follow-up of 13 months, atezolizumab had significantly improved OS compared with docetaxel (12.6 months vs. 9.7 months, p = 0.04). Increasing OS improvement was seen in subgroups with greater tumor cell and immune cell PD-L1 expression. In OAK trial, patients were randomly assigned (1:1) to intravenously receive either atezolizumab 1200 mg or docetaxel 75 mg/m^2^ every 3 weeks. OS was significantly longer with atezolizumab in the intention-to-treat (ITT) and PD-L1-expression populations. In the ITT population, OS was improved with atezolizumab compared with docetaxel (13.8 months vs 9.6 months; p = 0.0003). Patients in the PD-L1 low or undetectable subgroup also had improved survival with atezolizumab (12.6 months vs 8.9 months). Overall survival improvement was similar in patients with squamous or non-squamous histology. Significant OS benefit was achieved in both trials, in favor of atezoluzimab while even more benefit was documented in patients with greater PD-L1 expression.

### 3.4 Avelumab (Bavencio, Merck KGaA and Pfizer)

Fully humanized monoclonal IgG1 antibody against PD-L1. In 2017, it has been granted approval for the management of metastatic Merkel cell carcinoma (MCC) and locally advanced or metastatic urothelial carcinoma.

#### 3.4.1 Merkel Cell Carcinoma

In March 2017, avelumab was approved for the treatment of adults and pediatric patients 12 years and older with metastatic MCC, including those who have not received prior chemotherapy, based on the results of the JAVELIN phase 2 trial [75]. In this multicentre, international, prospective, single-group, open-label, phase 2 trial, patients with stage IV chemotherapy-refractory, histologically confirmed Merkel cell carcinoma were given intravenously at a dose of 10 mg/kg every 2 weeks. The ORR reached 31.8% (95.9% CI: 21.9–43.1), including 9.1% complete responses and 22.7% partial responses. The response lasted for more than six months in 86% of responding patients and >12 months in 45% of responding patients. Bavencio thereby received an accelerated approval, enabling to fill an unmet medical need using clinical trial data that is thought to predict a clinical benefit to patients.

#### 3.4.2 Urothelial Cancer

In May 2017, accelerated FDA approval was granted to avelumab for the treatment of patients with platinum-refractory metastatic UC based on the pooled analysis from two expansion cohorts of the open-label phase I trial dose-expansion JAVELIN Solid Tumor trial [76] (https://clinicaltrials.gov/ct2/show/NCT01772004). In this open-label, single arm, multi-center study, patients with UC progressing after platinum-based chemotherapy and unselected for PD-L1 expression received avelumab 10 mg/kg every 2 weeks. Confirmed ORR in patients who had been followed for at least 13 weeks was 13.3% (n = 30) (95% CI: 9.1–18.4), and 16.1% (95% CI: 10.8–22.8) in patients who had been followed for at least 6 months. Median time to response was 2.0 months (range 1.3–11.0). The median ROD had not been reached in patients followed for at least 13 weeks or at least 6 months.

### 3.5 Durvalumab (Imfinzi, AstraZeneca)

human immunoglobulin G1 kappamonoclonal antibody that blocks the interaction of PD-L1 with PD-1 and CD80. It is approved for use in locally advanced NSCLC as well as advanced UC.

#### 3.5.1 Urothelial Cancer

In May 2017, durvalumab was approved for the treatment of patients who have disease progression during or following platinum-containing chemotherapy, or whose disease has progressed within 12 months of receiving platinum-containing chemotherapy before (neoadjuvant) or after (adjuvant) surgery, based on data from Study 1108 [77,78]. In the trial, the ORR reached 17.0% (95% CI: 11.9–23.3) regardless of PD-L1 status, and 26.3% (95% CI: 17.8–36.4) in patients with PD-L1 high-expressing tumors. Additionally, approximately 14.3% of all evaluable patients achieved partial response and 2.7% achieved complete response. Based on a secondary endpoint in this single-arm trial, median time to response was six weeks. Among the total responding patients, 45% patients had ongoing responses of six months or longer and 16% patients had ongoing responses of 12 months or longer.

#### 3.5.2 NSCLC

In February 2018, based on the results of the phase 3 PACIFIC trial [79,80], durvalumab was approved for the treatment of patients with stage III NSCLC whose tumors are not able to be surgically removed (unresectable) and whose cancer has not progressed after treatment with chemotherapy and radiation (chemoradiation). In this trial, durvalumab improved median PFS by 11.2 months compared to placebo (16.8 vs 5.6 months; P < 0.001). The 12- month PFS rate was 55.9% (versus 35.3%) and the 18-month PFS rate was 44.2% (versus 27.0%) in favor of the anti-PD-L1 agent. The response rate was higher with durvalumab than with placebo (28.4% vs. 16.0%; P < 0.001), and the median DOR was longer (72.8% vs. 46.8% of the patients had an ongoing response at 18 months). The median time to death or distant metastasis was longer with durvalumab than with placebo (23.2 months vs. 14.6 months; P < 0.001).

### 3.6 Toxicities of PD-1/PD-L1 Signal Blocking

The side effects and immune related adverse events (IrAEs) associated with PD-1 blockade are generally considered to be well tolerated and manageable, particularly compared with the toxicity profile of CTLA-4 inhibitors [44,81,82] and chemotherapy. IrAEs of PD-1/PD-L1 inhibition include interstitial pneumonitis, colitis with gastrointestinal perforation, type 1diabetes, severe skin reactions, immune thrombocytopenia, neutropenia and sepsis after corticosteroid therapy, encephalopathy and neurological sequelae, Guillain-Barré syndrome, myelitis, myasthenia gravis, myocarditis and cardiac insufficiency, acute adrenal insufficiency, and nephritis [83]. One recent meta-analysis evaluated the safety and tolerability of PD-1/PD-L1 inhibitor in 3450 patients with advanced cancer from 7 randomized controlled studies [84]. Compared with chemotherapy, the PD-1/PD-L1 inhibitors had a significantly lower risk of all- and high- grade fatigue, sensory neuropathy, diarrhea and hematologic toxicities, all-grade anorexia, nausea, and constipation, any all- (67.6% versus 82.9%) and high-grade AEs (11.4% versus 35.7%), and treatment discontinuation (4.5% versus 11.1%). Although IrAEs are usually mild and could be managed by clinicians, there are some severe effects that may be deadly, such as pneumonitis, cardiorespiratory arrest, cardiac failure, myocardial infarction and stroke. In July 2017, the FDA has placed clinical holds on several clinical trials investigating pembrolizumab-, nivolumab-, and durvalumab-containing regimens in various hematologic malignancies due to the safety concerns originated from the KEYNOTE-183 and KEYNOTE-185 studies [85]. Prevention, early recognition (grade 1–2 adverse effects) and prompt intervention are of great importance in management of these severe cases and have the potential to prevent patient morbidity and mortality [86]. Notably, the use of immunosuppressive agents - essentially steroids - in the management of the side effects does not appear to compromise the checkpoint blockade efficacy [87]. Guidelines and specific care algorithms should be optimized for the identification, early intervention, and management of IrAEs.

## 4 Profiling the Factors Affecting Therapeutic Efficacy/Toxicity

Despite the promising anticancer activity offered by PD-1 and PD-L1 inhibitors, only a fraction of patients exhibit dramatic responses to single-agent anti-PD-L1/PD-1 antibodies treatment, mostly ranging from 15 to 35% depending on the individual's indication (exceptions include microsatellite-instable tumors, Merkel cell carcinoma and Hodgkin lymphoma, for which objective response rates are 50–80%). To determine why PD1 blockade is effective in some patients but not others, the molecular mechanisms underlying the resistance to checkpoint blockade should be elucidated [88] and a variety of factors that contribute to determining whether a response occurs need to be identified through more basic and clinical studies [1].

Most attention has been paid to PD-L1. PD-L1 can be expressed on tumor cells and tumor infiltrating immune cells (TIICs), particularly myeloid APCs (macrophages and myeloid DCs), mediating T cell suppression [28,89]. A correlation has been observed between the expression of PD-L1 in tumor tissue and the likelihood of the response to blockade therapy in various malignancies where pre-existing immunity is presumably suppressed by PD-L1 [71,73]. So far, there are 4 FDA approved assays of PD-L1 expression by immunohistochemistry to help guide treatment decisions for nivolumab in advanced NSCLC or melanoma, pembrolizumab in advanced NSCLC, atezolizumab in advanced UC or NSCLC, and durvalumab in advanced UC [44,58,77,90,91]. Additionally, PD-L1 can be adaptively expressed following exposure to IFN-γ [5,92,93], meaning the expression of PD-L1 on TIICs can reflect the activity of effector T cells [94]. One recent meta-analysis involving 3674 patients from 18 trials evaluating the correlation between PD-L1 expression in TIICs and the survival of cancer patients suggested PD-L1 positive expression on TIICs was correlated to a lower risk of death (HR = 0.784, 95% CI: 0.616–0.997, P = 0.047) [95]. However, the expression of PD-L1 in tumor tissues should not be used as criteria to exclude patients from the treatment with either anti-PD-1 or anti- PD-L1 antibodies, as patients whose tumors were stained as PD-L1 negative can have objective responses. For instance, melanoma patients can have a clinical response regardless of PD-L1 expression status. The expression of PD-L1 on the surface of tumor cells and immune cells before immunotherapy may be a useful but so far not a definitive predictive biomarker. Taken together, predicting tumor responses to PD-1/PD-L1 blockade remains a greatest challenge and considerable efforts should be made to profile the complex and dynamic factors governing the strength and duration of immune response in the immunotherapy, making treatment decisions on a personalized basis.

### 4.1 Tumor Mutation Burden and Neoantigens

Tumors can acquire thousands of different somatic mutations during transformation and progression. Genetic heterogeneity of tumor cells has been recognized as a fundamental property of cancers, where selective pressure may lead to the outgrowth of clones with superior fitness [6,7]. Somatic mutations can be categorized as passenger or driver mutations; passenger mutations represent the majority of mutations, having no particular effect on the fitness of the cell, whereas driver mutations typically make up only a small fraction of mutations per cell, however providing a critical selective advantage to malignant clones. Tumor mutation burden (TMB) is defined as the total number of mutations per coding area in a tumor genome and this may range from a few to thousands of somatic mutations [82]. Somatic mutations, such as point mutations, frame-shift mutations or insertion or deletion mutations regardless of driver or passenger status, may create new protein or peptide sequences called neoantigens that can be immunogenic due to the sequence divergence and lacking central tolerance.

Several lines of evidence suggest that high TMBs are associated with the enhanced responsiveness to PD-1 pathway blockade in some cancer patients. For instance, in patients with NSCLC treated with pembrolizumab, higher non-synonymous mutation burden in tumors was found to be associated with improved objective response, durable clinical benefit, progression-free survival, and neoantigen-specific CD8^+^ T cell responses paralleled tumor regression [96]. Similarly, analysis of whole-exome sequencing of colorectal cancers showed that high TMB load was correlated with increased numbers of TILs and improved survival [69]. High response rates have also been demonstrated in the virally induced Merkel cell carcinoma of the skin [97], desmoplastic melanoma that have high numbers of somatic mutations as a result of exposure to ultraviolet radiation [98]. In December 2017, FDA approved the only test known as FoundationOne test that can be utilized to assess the states of TMB in patients. This is a next-generation liquid biopsy test for solid tumors that been used in clinical trials to show that it can predict response to anti-PD-1/PD-L1 therapy in various cancer types [[99], [100], [101], [102]].

It is crucial to understand the immunogenicity ultimately determine whether given TMB-associated neoantigens are recognized as target by the immune system, and lack of high quality of neoantigens, impaired neoantigen processing, and/or impaired presentation of neoantigens may all lead to comprised response to checkpoint blockade, as T cells may not mount an attack against tumor cells. The mutations in the genes KRAS and BRAF or other mutations involved in the activation of the MAP kinase pathway will decrease the expression of MHC class I molecules or effect the molecules that are essential for peptide loading, resulting in insufficient anti-cancer immunity and the poor response in some patients even with high TMB [5,[103], [104], [105]].

However, measuring TMB requires whole-exome sequencing that is costly and time consuming to be applied as a standard clinical test [106]. Cancer gene panels composed of hundreds of cancer-related genes are investigated to profile tumor genetics. Recently, Johnson et al. showed the mutation counts detected in the 315-gene NGS panel for melanoma could be highly correlated with those assessed by whole-exome sequencing (Spearman coefficient = 0.995) [107]. Similarly, Roszik et al. employed approximately 170 genes in the NGS panels to develop an algorithmic method to estimate total mutation load within tumors and to predict the efficacy of immunotherapy [108]. Although these results indicate that the NGS gene panels with hundreds of genes can be used to assesse TMB as an independent factor in predicting response to immunotherapy, the cost of the NGS gene panels with >150 genes is still high for the routine clinical tests in most hospitals. Continuing to improve TMB estimating algorithms in cancer-specific manner may considerably decrease the cost and time required for the TMB assessment, representing a promising approach in response prediction to cancer immunotherapy.

### 4.2 Tumor Microenvironment

It is generally accepted that successful anti-tumor immune responses following PD-1/PD-L1 blockade require reactivation and clonal-proliferation of tumor-specific T cells present in the tumor microenvironment (TME) and the differences in the outcome of cancer immunotherapy can be partially attributed to the heterogeneity of the tumor microenvironment [109,110].

Two basic TME profiles can be distinguished: “hot” immune-inflamed TME that is associated with higher densities of CD^8+^ tumor-infiltrating lymphocytes (TILs), accompanied by myeloid cells and monocytic cells, higher levels of IFN and IFN stimulated chemokines such as CXCL9, CLCL10 and CXCL11 present and various other proinflammatory and effector cytokines may predict benefit from PD-1 blockade therapy. And indeed, clinical responses to anti-PD-L1/PD-1 therapy occur most often in patients with inflamed tumors. In contrast, non-inflamed tumors with “cold” TME generally express cytokines that are associated with immune suppression or tolerance, such as IL-10, IL-35, IL-4, TGF-β et al. [[111], [112], [113], [114]]. They can also contain cell types associated with immune suppression or tissue homeostasis, for instance myeloid-derived suppressor cells [[115], [116], [117]], inactivated M2 macrophages [118,119], regulatory T_reg_ cells. Accordingly, in the absence of pre-existing antitumor immunity such tumors usually respond poorly to anti-PD-L1/PD-1 therapy.

However, why some tumors are “inflamed” with effector T cell infiltration, whereas others are not, remains to be elucidated. It appears “inflamed” tumors have characteristic immune profiles or signatures that are profoundly governed by tumor genetics and epigenetics. As mentioned previously, the greater the number of mutations in a given tumor, the more probable it is that some of the mutations will be immunogenic, providing targets for T-cell attack. But if the mutations negatively affect the antigen processing, antigen presentation and recognition or immune cells migration, they would cause malfunctioned immunity, resulting in non-inflamed TME [120]. For example, loss of B2M expression reduced the cell surface expression of MHC class I, thereby impairing antigen presentation to cytotoxic T cells; PTEN deficiency was associated with increased levels of CCL2 and VEGF, partially diminishing the infiltration of T cells; alterations in Wnt/β-catenin signaling caused decreased CCL4 production, which led to diminished infiltration of CD^103+^ dendritic cells [103,104]. Further more, epigenetic mechanism has also been implicated in creating non-flamed TME and epigenetic silencing of immune-related genes could be a key mechanism of tumor immune escape. It has been reported that polycomb repressive complex 2 (PRC2) [121,122], the demethylase JMJD3-mediated histone H3 lysine 27 trimethylation (H3K27me3), and DNA methylation repress the expression of Th1-type chemokines, such as CXCL9 and CXCL10, and subsequently restrain effector T cell infiltrating into TME. And epigenetic reprogramming may unlock the repression of Th1-type chemokine secretion, IFN signature genes expression and tumor antigen expression, therefore conditioning tumor from poor T cell infiltration to rich T cell infiltration might ultimately potentiate PD-1 blockade therapy [81,91].

### 4.3 Microbiome

The composition of gut microbiome has emerged as one of major factors that exert profound impact on an individual's potential to respond to immune checkpoint inhibitors [123]. It has been shown that the efficacy of cancer immunotherapy with immune checkpoint antibodies can be diminished with administration of antibiotics, and superior efficacy is observed with the presence of specific gut microbes, such as *Bifidobacteria* spp., *Akkermansia muciniphilia*, *E. hirae*, and *Bacteroides* spp., among others [[124], [125], [126], [127]]. Similarly, based on retrospective multivariate analysis, the receipt of antibiotics prior to immunotherapy was a negative predictor of survival. The composition of putatively favorable to unfavorable bacteria between responders and non-responders to anti-PD-1 therapy has been analyzed. For metastatic melanoma, the enrichment of the *Ruminococcaceae* family of the *Clostridiales* order was revealed in responders, in contrast to that the *Prevotellaceae* family of the *Bacteroidales* order enriched in non-responders [85]. It has even been proposed to employ bacteriophages as highly selective tool to specifically eliminate unfavorable bacteria as a potential intervention tool to enhance the efficacy of immunotherapy.

## 5 Summary and Outlook

Because of the complexity of immuno-regulatory mechanisms and the heterogeneity of malignancies, combination therapies represent the next wave of clinical cancer treatment that enable to overcome the limitations associated with single-agent therapy [17,128]. The PD-1 pathway blockade that has elicited durable clinical responses in a subset of patients largely relies on efficient T cell infiltration and effector T cells function in TME. Therefore, for rational combination therapies, it is important to consider how treatments converge to influence the antitumor immune response and the tumor itself. So far multiple abnormalities differentiating cancer cells from normal cells are suggested to be targeted in combination therapy, including reducing tumor burden and increasing tumor immunogenicity (such as to combine with chemotherapy, radiotherapy and targeted therapy); enforcing effector T cell trafficking with epigenetic reprogramming drugs (such as using EZH2 inhibitor 3-Deazaneplanocin A (DZNep), GSK126 and DNMT inhibitor 5-AZA-dC); blocking other inhibitory receptors, such as lymphocyte-activation gene 3 (LAG3), T-cell immunoglobulin and mucin-domain containing-3 (TIM3) [129,130]; interfering gut microbiome prior to the treatment; delivering agonists for co-stimulatory molecules; vaccination to boost T cell responses [131] and delivering effector T cells through adoptive T cell therapy.

In addition to focusing on stimulating adaptive T cell mediated elimination of tumor, targeting innate immune system could be a promising strategy. Innate immune cells, such as macrophages, NK cells, neutrophils and other myeloid cells play an important role in complementing the effector activities of T cells and can be recruited in large numbers from the circulation or TME to bolster an ongoing adaptive response. Various combination treatments have now been under investigation. For instance, immunotherapies combining targeting CD47/signal-regulatory protein alpha (SIRPα), an innate anti-phagocytic axis between tumor cells and macrophages were shown to elicit synergistic anti-cancer activities in both hematologic malignancies and solid tumors [[132], [133], [134]]. Or with the anti-CD52 antibody alemtuzumab, both neutrophils and NK cells were shown to be capable of effectively exerting antibody-dependent cellular cytotoxicity (ADCC) on CD52-expressing tumor cells [135]. Briefly, there is a growing appreciation of the potential contributions of innate immune effectors to anti-tumor immunity and integrating a variety of means targeting adaptive immune system into PD-1/PD-L1 blockade based therapies could be a very important combination approach in future immunotherapy.

The pace of cancer immunotherapy clinical studies is outstripping the progress in its basic research [5], which not only creates an opportunity to combine emerging scientific and clinical insights to deepen our understanding of cancer immunity but also presents a great challenge of establishing the guidance for future cancer immunotherapy. With the advancement of genomic, transcriptomic and immune profiling, a better understanding of molecular mechanisms underlying clinical successes versus failures will lead to the development of an integrative algorithm that may incorporate multiple factors to predict single agents or combination therapies that will work best for specific patients, thus leading us to an era of precision medicine or tailored immunotherapy.
